# The Influence of Micronutrients and Environmental Factors on Thyroid DNA Integrity

**DOI:** 10.3390/nu17132065

**Published:** 2025-06-21

**Authors:** Katarzyna D. Arczewska, Agnieszka Piekiełko-Witkowska

**Affiliations:** Department of Biochemistry and Molecular Biology, Centre of Translational Research, Centre of Postgraduate Medical Education, 01-813 Warsaw, Poland; agnieszka.piekielko-witkowska@cmkp.edu.pl

**Keywords:** thyroid, DNA damage, DNA repair, selenium, iodine, iron, zinc, copper, vitamins, environmental factors

## Abstract

Micronutrients and environmental factors are key exogenous agents influencing thyroid DNA integrity. Micronutrients act as cofactors in DNA replication, repair, and antioxidant defence, while environmental exposure, such as radiation, heavy metals, and endocrine-disrupting chemicals, can directly damage DNA, leading to genomic instability. Although many studies have confirmed the link between micronutrient status and thyroid health, the effects of nutrient imbalances and environmental stressors on thyroid DNA remain underexplored. This narrative review examines how these factors may compromise thyroid genome stability and contribute to disease development. The analysis focused on the roles of iodine, selenium, iron, zinc, copper and vitamins D, B9, and B12 as well as environmental exposures such as radiation, heavy metals, and endocrine-disrupting chemicals. The findings suggest that both micronutrient imbalance and environmental stress can impair DNA integrity in thyroid cells. Understanding these complex relationships is critical for developing effective strategies to maintain thyroid health and mitigate the risk of thyroid diseases associated with compromised genomic integrity. Methodology: This narrative review was based on 254 articles retrieved through a manual search of the PubMed and Google Scholar databases, covering the years 2000–2025 and focusing on the influence of micronutrients and environmental factors on thyroid DNA integrity and repair. Several seminal earlier publications, fundamental to a comprehensive understanding of the topic, were also included.

## 1. Introduction

Thyroid health influences the physiological status of the whole organism by regulating energy and calcium homeostasis through the synthesis and secretion of the thyroid hormones (TH) 3,5,3′-triiodothyronine (T_3_) and 3,5,3′5′-tetraiodothyronine (thyroxine, T_4_) as well as calcitonin. Thyroid hormones are master regulators of metabolism, growth, and development by controlling gene expression throughout the whole organism [1].

Dysfunctions of the thyroid gland are one of the most common disorders affecting humans worldwide [2]. Hypo- and hyperthyroidism predominantly result from autoimmune thyroid disease (AITD), but may also be associated with dysfunctions of the hypothalamic–pituitary–thyroid (HPT) axis, congenital diseases (e.g., thyroid dysgenesis or dyshormonogenesis), inappropriate iodide dietary supply, or pharmaceuticals interfering with TH synthesis (e.g., amiodarone, lithium, immune checkpoint inhibitors, iodine-containing compounds like iodinated contrast agents or radioiodine based diagnostic agents etc.) [3,4,5,6]. Although thyroid dysfunction usually does not pose a direct threat to human life (with the exception of rare thyroid emergencies [7]), it can lead to serious life-threatening disorders such as heart disease or hypercholesterolemia [8,9]. Thyroid deficiency is particularly dangerous during pregnancy and can lead to severe complications including developmental and births defects or an increased risk of stillbirth or miscarriage [10]. The thyroid gland can also be affected by neoplasia. Although most thyroid cancer types have a good prognosis, a rare anaplastic thyroid cancer is one of the most fatal, incurable malignancies [11].

Compared with other tissues, thyroid DNA is particularly exposed to endogenous damaging signals, since H_2_O_2_ production is required for the synthesis of thyroid hormones [12,13,14]. To protect their DNA, thyroid cells have complex defence mechanisms including antioxidant and DNA repair systems [15,16]. The disruption of DNA repair mechanisms or antioxidant defence may contribute to the development of various thyroid pathologies including autoimmune diseases and cancer. For example, polymorphisms in DNA repair genes modulate thyroid cancer (TC) susceptibility in general as well as an increase in radiation-related TC risk [16,17].

**Figure 1:**
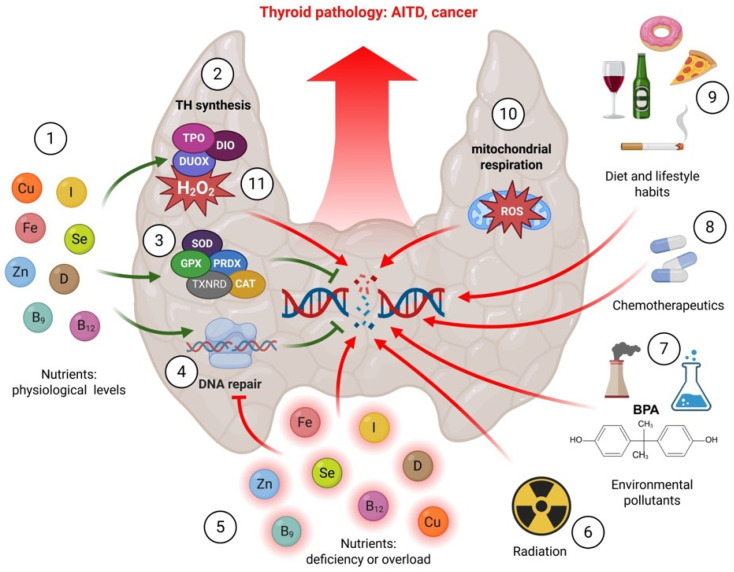
Micronutrients and environmental factors shape thyroid DNA integrity, thus influencing thyroid pathology including autoimmune thyroid disease (AITD) and cancer. (**1**) Adequate levels of micronutrients, such as iodine, selenium, zinc, iron, copper, and vitamins, maintain thyroid DNA integrity and physiology through regulating (**2**) thyroid hormone (TH) synthesis as well as supporting (**3**) anti-oxidative stress (OS) defence and (**4**) DNA repair and anti-oxidative stress (OS) defence systems. On the other hand, (**5**) unbalanced micronutrient levels may compromise DNA repair and OS defence or lead to DNA damage, thus compromising thyroid genome stability. Thyroid DNA integrity is threatened by exogenous (such as (**6**) radiation, (**7**) endocrine disrupting chemicals, (**8**) chemotherapeutics as well as (**9**) smoking, dietary and other lifestyle habits) or endogenous (including reactive oxygen species (ROS) generated as (**10**) by-products of mitochondrial respiration or (**11**) thyroid hormone (TH) synthesis). It should be noted that the trace elements depicted in the figure are presented in a symbolic manner. In reality, trace elements are consumed by mammals in their ionic forms (e.g., I^−^ or IO_3_^−^ in case of iodine, non-heme iron from plant sources) or as covalently bound components of organic complexes (e.g., heme iron from animal sources). Created in BioRender. Arczewska, K. (2025) https://BioRender.com/9lupn7y (accessed on 16 June 2025).

Important exogenous agents shaping thyroid DNA integrity include micronutrients as well as environmental factors. Micronutrients act as cofactors for enzymes involved in DNA replication and repair as well as antioxidant defence systems [18]. Conversely, environmental factors such as exposure to radiation, pollutants, and certain chemicals can directly damage the DNA structure, causing mutations and genomic instability [19]. The hypothesis on the associations between micronutrients and thyroid health has been verified by multiple studies (e.g., [20,21]). In contrast, the effects of inappropriate nutrient supply and the state of thyroid DNA have been less commonly addressed. Therefore, in this review, we aimed to comprehensively analyse published reports in the search for data indicating the influence of specific micronutrients (i.e., iodine, selenium, iron, zinc, copper, vitamins D, B9, and B12) as well as environmental factors (i.e., radiation, heavy metals, and endocrine disrupting chemicals) on thyroid DNA integrity (Figure 1). We discuss how a deficiency or excess of these micronutrients as well as exposure to the above environmental factors can potentially induce DNA damage or instability in thyroid cells, and as a consequence, lead to thyroid disease. Understanding the influence of the above factors on thyroid DNA integrity is of critical importance for developing effective preventive strategies and therapeutic interventions aimed at maintaining thyroid health and mitigating the risk of thyroid diseases.

## 2. Methodology

This narrative review was based on a manual search of the PubMed and Google Scholar databases for articles published between 2000 and 2025, supplemented by several seminal earlier publications essential for an understanding of the topic. The search focused on the relationships between thyroid diseases, DNA repair mechanisms, micronutrients, endocrine-disrupting chemicals (EDCs), and radiation. The following keywords and their combinations were used with Boolean operators (AND, OR): “thyroid”, “thyroid gland”, “thyroid diseases”, “thyroid dysfunction”, “thyroid cancer”, “hyperthyroidism”, “hypothyroidism”, “Graves’ Disease”, “Hashimoto Thyroiditis”, “autoimmune thyroid disease”, “thyroid nodules”, “goitre”, “DNA repair”, “DNA damage response”, “genomic stability”, “double-strand break repair”, “base excision repair”, “nucleotide excision repair”, “mismatch repair”, “oxidative DNA damage”, “micronutrients”, “trace elements”, “copper”, “zinc”, “selenium”, “iodine”, “vitamin D”, “folate”, “folic acid”, “vitamin B9”, “vitamin B12”, “cigarette smoke”, “endocrine disrupting chemicals”, “EDCs”, “bisphenol A”, “perfluoroalkyl substances”, “polyfluoroalkyl substances”, “radiation”, “ionising radiation”, “reactive oxygen species”, “ROS”, “oxidative stress”, “oxidative stress response”, “genomic stability”, “thyroid hormones”, and “thyroid hormone signalling”. Only peer-reviewed original and review articles published in English were considered. The reference lists of the selected papers were also screened to identify additional relevant sources. A total of 254 articles meeting the inclusion criteria were selected for analysis.

## 3. Micronutrients

Micronutrients (including iodine, selenium and iron) and environmental factors have a large impact on thyroid physiology and disease, contributing to TH biosynthesis, the thyroid oxidative state, immunity, and carcinogenesis. It needs to be underlined that most scientific papers use the colloquial terms of “iodine”, “selenium”, and “iron” while referring in fact to the “iodide”, “selenide”, and compounds containing ferrous/ferric ions [22]. Therefore, the reader should bear in mind that in fact, most studies cited hereafter refer to the iodine, selenium, and iron compounds, and not the elements in their atomic forms.

### 3.1. Iodide

**Figure 2:**
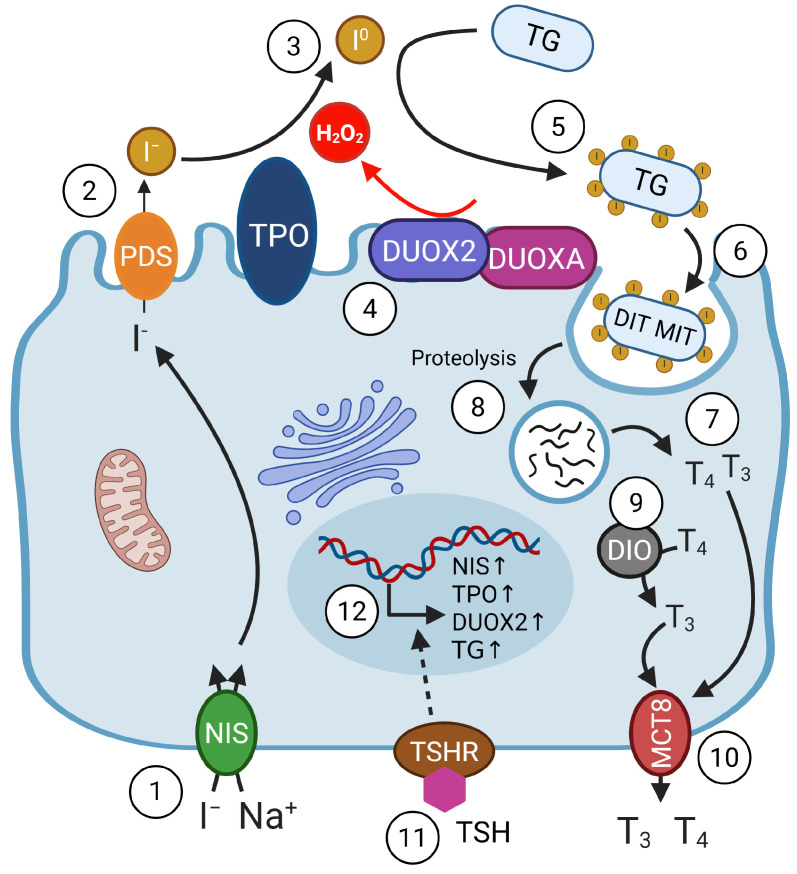
Thyroid hormone synthesis and secretion. Generation of the thyroid hormones (THs) triiodothyronine (T_3_) and tetraiodothyronine (T_4_) begins with the (**1**) import of iodide (I^–^) *via* the basolateral sodium/iodide symporter (NIS), which is followed by (**2**) iodide export to the thyroid follicle lumen by the apical anion exchanger pendrin (PDS; SLC26A4). In the follicle lumen, iodide is (**3**) oxidised to iodine radicals (I^0^) *via* a TPO-mediated, H_2_O_2_-dependent mechanism. H_2_O_2_ is primarily generated by (**4**) dual oxidase 2 (DUOX2), supported by its maturation factor (DUOXA2). Iodine radicals serve to iodinate tyrosine residues in (**5**) thyroglobulin (TG), ultimately producing (**6**) monoiodotyrosine (MIT) and diiodotyrosine (DIT). The coupling of MIT and DIT leads to the formation of (**7**) T_3_ and T_4_, which are stored in the follicle lumen and released into circulation upon TSH stimulation after endocytosis and (**8**) proteolytic processing. (**9**) Deiodinases DIO1 and DIO2 are responsible for converting T_4_ to the more potent T_3_. Finally, thyroid hormones are (**10**) transported out of the thyrocyte into the bloodstream by the MTC8 (SLC16A2) transporter. TH synthesis is stimulated by (**11**) TSH binding to its receptor (TSHR) and the downstream (**12**) upregulation of NIS, TPO, DUOX2, and TG. Created in BioRender. Arczewska, K. (2025) https://BioRender.com/ikhxakb (accessed on 19 June 2025).

Among all of the human organs, the thyroid accumulates the highest iodide content due to its ability to uptake iodide (I^−^) from the bloodstream against the concentration gradient through the activity of the NIS transporter (sodium/iodide symporter; SLC5A5) located at the basolateral membrane of the thyrocyte (see Figure 2) [23]. Followed by the uptake, the iodide is transported to the thyroid follicle lumen through the apically localised electroneutral anion exchanger PDS (pendrin; SLC26A4). In the thyroid follicle lumen, I^−^ ions undergo oxidation in a reaction mediated by thyroid peroxidase (TPO) and is dependent on H_2_O_2_ (generated by dual oxidase 2; DUOX2), resulting in the formation of iodine radicals (I^0^). This facilitates the iodination of tyrosine residues in thyroglobulin (TG) to yield diiodotyrosines (DITs) and monoiodotyrosines (MITs). DIT and MIT are further coupled in a TPO-catalysed reaction to form TH. The iodinated TG is sequestered within the follicular lumen; however, upon TH deficiency, TG is internalised by thyrocytes through endocytosis and subsequently undergoes proteolytic degradation within the endolysosomes, resulting in the liberation of T_3_ and T_4_. TH is then translocated from the thyrocyte into the bloodstream via monocarboxylate transporter 8 (MTC8; SLC16A2) [3,12,13].

Iodide, apart from being essential for TH production, also affects the functioning of thyroid cells. Iodide is an ancestral antioxidant whose function is widely conserved from algae to vertebrates [24]. It acts as a ROS-scavenger and inhibitor of inflammatory response, minimising oxidative stress (OS)-induced damage and lipid peroxidation. Iodide’s antioxidative capacity is additionally manifested through the induction of NRF2 (erythroid 2-related transcription factor 2, also known as nuclear factor erythroid-derived 2-like 2; NFE2L2) transcriptional activity. This occurs via the iodination-dependent dissociation of NRF2 from its cytoplasmic repressor, KEAP1 (Kelch-like ECH-associated protein 1 (KEAP1), facilitating the subsequent nuclear translocation of NRF2, as demonstrated in human keratinocytes and skin tissue. According to some studies, iodide may exert anticancer activity by attenuating proliferation and facilitating apoptosis. Furthermore, it inhibits the formation of mutagenic oestrogen-DNA adducts, as revealed in several cancer cell and animal models including thyroid cells [25,26]. However, other studies suggest that excessive iodide intake may contribute to the development of PTC (further discussed below).

Iodide deficiency at the developmental stage leads to endemic cretinism, while in adults, it increases the risk of thyroid nodules, goitre, AITD as well as thyroid cancer. The suggested pro-neoplastic effects of iodide deficiency include chronic thyroid-stimulating hormone (TSH)-mediated stimulation of thyrocyte proliferation, which is associated with elevated DNA damage [27,28,29,30,31]. Additionally, iodide deficiency also upregulated *Nis* and *Tpo* expression, induced ROS production (presumably through increased Duox2 activity) and the resulting oxidative DNA damage, and increased the expression of the antioxidative defence enzymes superoxide dismutase 3 (*SOD3*), glutathione peroxidase 4 (*GPX4*), and peroxiredoxins 3 (*PRDX3*) and 5 (*PRDX5*), but had no influence on the somatic mutation rates in the thyroids of the experimental rats and mice [32,33]. On the other hand, adequate iodide levels are suggested to reduce the dedifferentiation of thyroid tumours and lower the follicular thyroid cancer (FTC) and ATC incidence, the action that might, at least partially, be exerted by preventing the downregulation of thyroid differentiation markers TPO, NIS, and PDS [25,30,34]. Additionally, low iodide concentrations upregulated the expression of *Tpo* and several antioxidative defence genes in normal murine thyrocytes, but they failed to do so in thyrocytes derived from NOD-H2^h4^ mice, a mouse model exhibiting a pronounced susceptibility to iodine-induced autoimmune thyroiditis [35].

On the other hand, excessive iodide levels lead to cell death, specifically in thyroid cells [36,37]. Iodide overload also increases AITD and cancer incidence [30,31]. Moreover, excessive iodide levels downregulate the expression of *DUOX2*, *TPO*, *TG*, and *NIS*, resulting in the attenuation of the TPO-mediated iodination of TG and the biosynthesis of TH, a phenomenon referred to as the Wolff–Chaikoff effect [6,14]. Apart from TH synthesis downregulation through the Wolff–Chaikoff effect, iodide also compromises thyrocyte proliferation and survival. It has been suggested that excessive iodide acts through elevated ROS production and the generation of oxidatively-modified iodolipids (6-iodo-lactone and 2-iodo-hexadecanal). The escape from the Wolff–Chaikoff effect, on the other hand, which leads to the restoration of TH synthesis and accompanied re-expression of NIS and TPO, results from the upregulation of the antioxidative stress response [30,38,39,40]. Interestingly, thyroid cancer diagnosis using radioactive-iodine based agents may lead to a phenomenon known as thyroid stunning, where secondary to the diagnostic dose application, an inhibition of the therapeutic radioiodine accumulation in the thyroid is observed [40]. It has been proposed that thyroid stunning results from DNA damage-mediated and DNA damage sensor ATM kinase-dependent downregulation of NIS expression [41,42,43]. Additionally, an iodide excess in the AITD murine model induced DNA damage, mediated through downregulation of the Mth1 protein [44]. MTH1, namely human MutT homologue 1 (aka NUDT1) prevents oxidative DNA damage through inhibiting the incorporation of oxidatively modified nucleotides, and MTH1 is required for maintaining malignant traits of thyroid cancer cells [45,46]. Another aspect related to thyroidal iodide overload that has never been studied is direct DNA modification leading to the generation of iodinated nucleic base derivatives (e.g., 8-iodo-2′-deoxyguanosine (8-I-dG)), which similarly to the chlorinated and brominated derivatives might potentially be mutagenic [38].

In conclusion, both an insufficient and excessive iodide supply can increase the risk of thyroid disorders. This indicates that maintaining an adequate iodide balance is essential for the synthesis of thyroid hormones, the preservation of thyroid DNA integrity as well as the control of thyrocyte proliferation and apoptosis.

### 3.2. Selenium

The thyroid contains the highest selenide (Se) levels among all the tissues in the body [47,48]. Selenide is required for protection against OS and for thyroid hormone production, since it is essential for selenoprotein synthesis, where it is incorporated co-translationally as the active site residue selenocysteine. The major selenoproteins involved in thyroid homeostasis are the antioxidative defence proteins glutathione peroxidases (GPXs: GPX1-GPX8) and thioredoxin reductases (TXNRDs/TRXRs: TXNRD1/TRXR1-TXNRD3/TRXR3) as well as iodothyronine deiodinases (DIO1-3) [49].

GPX1 prevents the excessive TPO-dependent iodination of thyrocyte intracellular proteins and is upregulated in TC tissues [46,50,51]. Among other GPXs, GPX3 is downregulated, whereas GPX4 is upregulated in thyroid cancer tissues. GPX4 is the only GPX essential in mice and is required for iron-dependent cell death, ferroptosis [51,52]. TXNRDs are required for reducing disulphide bonds in a variety of thiol-dependent proteins maintaining genome stability, among them ribonucleotide reductase, apurinic/apyrimidinic endonuclease 1 (APE1), and TP53 [53]. Leoni et al. demonstrated that selenium increases TSH-induced NIS expression by regulating the DNA binding activity of transcription factor Pax8. This process is critically dependent on the redox activity of Ape1, which reduces Pax8, and this reduction is maintained by TxnRd1 in thyroid rat cells [54]. Interestingly, cytoplasmic TXNRD1 is downregulated in TC tissues, whereas mitochondrial TXNRD2 is upregulated in TC and multinodular goitre thyroid tissues [55,56].

Among the other selenoproteins, *DIO1*, *DIO2*, *SELENOO*, *SELENOP*, *SELENOS*, and *SELENOV* are downregulated in human TC tissues [57]. On the other hand, SELENBP1, which is not selenoprotein, but intracellular protein able to bind selenium that protects cells against oxidative stress, upregulates p53 and is considered as a tumour suppressor in multiple cancer tissues [58]. Surprisingly, in TC, SELENBP1 is upregulated and supports malignant traits of thyroid cancer cells [59]. Selenoprotein H (SELENOH/SELH) is particularly interesting in the context of genomic stability. SELENOH localises to the nucleus and binds DNA, thereby preventing nuclear oxidative stress [60]. Although specific information regarding SELENOH expression in the thyroid is limited, an analysis of the Protein Atlas (https://www.proteinatlas.org/, accessed on 23 April 2025) and GTEx (https://gtexportal.org/, accessed on 23 April 2025) databases suggest its moderate to high expression in a normal thyroid. Intriguingly, the levels of SELENOH and TXNRD as well as oxidatively-modified DNA lesion 8-oxo-7,8-dihydroguanosine (8-oxoGuo) were elevated in the serum samples of Hashimoto thyroiditis (HT) patients [61].

The role of DIO1 and DIO2 in the thyroid and extrathyroidal tissues is to convert T4 to the more potent T3, whereas DIO3 is responsible for TH inactivation [62]. In papillary thyroid cancer (PTC), DIO1 and DIO2 mRNA and activity are typically decreased compared with normal tissue [63]. On the other hand, DIO3 is upregulated in PTC tissues, and elevated DIO3 levels are associated with the BRAF^V600E^ mutation, larger tumour size, and metastasis [64,65]. This pattern of high DIO3 and low DIO1/DIO2 in PTC might be related to dedifferentiation and suggests a local hypothyroid environment that may support minimal growth and contribute to invasiveness [66]. In contrast, aggressive anaplastic thyroid carcinoma (ATC) exhibits a distinct deiodinase profile with high DIO2 expression and significantly lower DIO1 and DIO3 levels compared with PTC and normal cells. This leads to potentially increased intracellular T3 and enhanced TH signalling in ATC, which appears crucial for maintaining the aggressive cancerous phenotype; notably, inhibiting DIO2 in ATC cells reduces growth, induces senescence, and decreases invasive potential, suggesting DIO2 is a potential therapeutic target in this late-stage cancer [67]. While expression patterns in follicular thyroid carcinoma (FTC) and follicular thyroid adenoma (FTA) are less consistent across studies, some have reported increased DIO1 and/or DIO2 expression in FTA/FTC, and DIO3 was detected in FTC but showed lower levels than in PTC [63,66]. Interestingly, the connection between genome stability and TH production can be exemplified by progeroid mice deficient in nucleotide excision DNA repair that revealed hypothyroidism with preserved normal thyroid morphology. The above phenotype is thought to result from the accelerated ageing-accompanied reduction in DIO1 activity and rise in DIO3 activity [68]. Selenium deficiency leads to reduced DIO activity inducing the hypothyroidic state, which in turn stimulates TSH production and subsequently exacerbates thyroidal H_2_O_2_ generation. Subsequently, increased H_2_O_2_ levels are confronted with the reduced GPX and TXNRD activity in the thyroid cell. This in turn potentiates OS, DNA damage, and cell death, and as a consequence leads to thyroid inflammation and fibrosis [69,70,71].

In mouse models, selenide deficiency influences, to the greatest extent, the expression of the so-called stress-related selenoproteins, including Dio1 and Gpx1, whereas housekeeping selenoproteins, such as Dio2, Gpx4, Txnrd1-3, are almost unaffected [72]. Conversely, supplementation with Se-containing compounds exerts anti-OS effects, as evidenced by the increase in the Gpx1, TxnRd1, and Prdx5 levels in the murine AITD model [73]. Moreover, selenium compounds protect human thyrocytes from oxidative DNA damage [74,75]. Studies in other cell systems suggest that this protection might be conferred by the induction of p53- and CHK2-dependent DNA repair pathways [76,77]. Interestingly, ALKBH8, one of the α-ketoglutarate-dependent dioxygenase AlkB family proteins repairing DNA and RNA alkylation damage, methylates the wobble uridine, which is required for the generation of several tRNAs including selenocysteine tRNA (tRNA^Sec^). *Alkbh8^−/−^* knockout mice and MEFs have reduced selenoprotein levels, but without the reported thyroid-specific phenotype [78,79]. Moreover, truncating ALKBH8 mutations in humans lead to intellectual disability, again without thyroid manifestation [80]. On the other hand, mice with selenoprotein-deficient thyroids, generated by conditional disruption of the tRNA^Sec^ genomic locus, revealed a normal thyroid gross morphology and reduced thyroidal Dio1 and Gpx1 levels accompanied by increased OS and elevated serum TSH, but without a change in TH levels. This suggests that although selenide is important for thyroid homeostasis, it is not essential for thyroid integrity [81]. However, notably in humans, defective selenoprotein synthesis due to mutations in tRNA^Sec^ or SECISBP2 (SECIS binding protein 2) genes leads to an altered thyroid morphology and abnormal serum TH levels [82]. Additionally, severe selenide deficiency, leading to endemic Keshan or Kashin–Beck diseases, manifested through cardiomyopathy and degenerative osteochondropathy, respectively, is not associated with a thyroid-specific phenotype. However, the combination of severe selenide and iodide deficiency in children leads to myxedematous cretinism, characterised by iodide supplementation-refractory hypothyroidism [71].

Selenide deficiency has been observed in nodular goitre and thyroid cancer patients and was suggested as a risk factor in TC, but not all studies confirmed such an association. On the other hand, selenide supplementation might protect from thyroid tumour onset through its anti-OS and anti-DNA damage activities. Additionally, selenide was suggested to have beneficial effects in TC management, as it was observed to influence multiple malignant traits of the cancer cells, induce cancer cell death, and inhibit thyroid tumour cell growth in a xenograft murine model [70,83,84,85,86]. In line with these observations, human thyroid cancer cell lines responded to selenide treatment by cell cycle arrest in the S and G_2_/M phases, which was accompanied by the upregulated expression of DNA damage response factors GADD34/PPP1R15A and GADD153/DDIT3 [87]. However, the clinical utility of selenide supplementation in TC still requires validation.

Selenide is also described as an immunomodulator, which together with its thyroid integrity protecting anti-OS activity, constitutes the basis for the observed selenide deficiency implication in autoimmune thyroid diseases [49,70,71,84,88]. As a consequence, multiple studies have tried to correlate selenide supplementation with AITD amelioration, but currently, the results of these studies are in general inconclusive and do not allow for a recommendation of selenide in these disease states. The only proven beneficial effect of selenide supplementation in AITD was observed in patients with mild thyroid eye Disease (TED)/Graves’ orbitopathy (GO), leading to the recommendation of selenide supplementation in TED patients [49,70,86,89,90].

### 3.3. Iron

Iron cations play a complex role in multiple cellular processes involving oxidation–reduction reactions including mitochondrial respiration, biogenesis of amino acids, ribosomes, and tRNA as well as DNA replication and repair, proliferation, and cell death throughout the whole organism. The dark side of oxidoreductive iron properties is the induction of oxidative stress through hydroxyl radical generation in Fenton-type reactions [91,92]. Accordingly, Fenton reaction constituents induce oxidative damage in both genomic and mitochondrial DNA in porcine thyroid homogenates [93,94]. The iron is essential for mitochondrial energy production, where it plays a crucial role as a component of iron-sulphur (Fe-S) clusters (ISCs) as well as heme prosthetic groups within the electron transport chain (ETC) proteins. Iron is also required by other mitochondrial enzymes that participate in the citric acid cycle and other metabolic pathways. Importantly, within mitochondria, some essential steps of the biogenesis of ISCs present in all cellular iron-sulphur cluster-containing proteins takes place [95]. Importantly, several proteins involved in ISC synthesis revealed an altered expression in cancer tissues including thyroid cancer. Most notably, among those upregulated in TC tissues were terminal enzymes of the cytosolic iron-sulphur cluster assembly (CIA) pathway, namely CIAO1 (cytosolic iron-sulphur assembly component 1), CIAO2B (cytosolic iron-sulphur assembly component 2B), and MMS19/MET18 (methyl-methanesulfonate sensitivity 19), which form a complex inserting Fe-S cluster into the recipient proteins [96]. MMS19 directly interacts with several ISC-containing DNA repair proteins, and its deficiency leads to genomic instability [97,98]. Multiple enzymes that are fundamental for DNA replication and stability frequently utilise iron within the structure of Fe-S clusters or heme/non-heme iron groups at their catalytic centres (Table 1).

Both iron deficiency and overload can lead to mitochondrial dysfunction, accompanied by the disruption of iron-sulphur cluster biogenesis. This leads to a disturbance in DNA synthesis or repair, thereby resulting in the increased DNA damage. These findings indicate that keeping the iron levels within a physiological range is vital for maintaining genome stability [135,136,137]. Importantly, TPO and NOX oxidases, including DUOXs, contain heme iron prosthetic groups, therefore iron homeostasis is essential in thyroid health and for TH synthesis [13,138,139,140]. A deficiency of iron cations or anaemia is frequently observed in AITD and hypothyroid patients, which is suggested to result from their malabsorption in the intestine or elevated bleeding due to the frequent co-occurrence of gastrointestinal tract autoimmune disease or from the genital system in women, or reduced erythropoiesis as a consequence of TH deficiency [88,139,141,142,143]. Conversely, iron overload, such as in genetic hemochromatosis or beta-thalassemia, have also been associated with an increased risk of autoimmune thyroiditis and hypothyroidism. In beta-thalassemia, hypothyroidism might result from both direct iron overload-induced thyroid gland destruction as well as from Fe accumulation in the pituitary, leading to central hypothyroidism [144,145]. However, the field suffers from a scarcity of studies directly examining the impact of varying iron levels on thyroid nuclear DNA integrity.

Cancer cells, including thyroid cancer cells, depend on a high supply of Fe ions, which are required for maintaining an elevated metabolism and proliferation rate as well as to support metastatic potential [146]. To satisfy these demands, TC cells have increased expression of transferrin receptor (TFR1), which enables a sufficient iron supply via transferrin, the major transporter of iron ions in human blood. The other mechanisms supporting iron supply in TC include the upregulation of lipocalin-2 (LCN2/NGAL), the innate immune response protein, which delivers iron cations to cancer cells, the downregulation of ferroportin (FPN1), which is responsible for efflux of iron ions from the cell as well as the upregulation of hepcidin, the secreted protein that promotes FPN1 degradation [91,147]. Importantly, TFR1 supports malignant traits of cultured TC cells [148]. Moreover, the suppression of hepcidin expression and secretion through downregulation of the E4BP4/G9a/SOSTDC1/hepcidin pathway or LCN2 silencing inhibits TC growth in vitro and in vivo [149,150]. Interestingly, as shown in other cell systems, both intracellular iron depletion through FPN1 upregulation or the application of iron chelators as well as iron overload through iron or transferrin treatment induce DNA damage [135,151,152,153,154]. In normal cells, the excess of iron cations triggers the iron-dependent cell death pathway known as ferroptosis. Interestingly, DNA damage response (DDR) proteins, including ATM and ATR as well as p53, were implicated in ferroptosis, however, without DNA damage involvement [155]. Tumour cells have evolved mechanisms to avoid ferroptotic cell death [146]. Importantly, a ferroptosis-related gene signature-based algorithm, including both protective and risk factors, was proposed to predict PTC prognosis, and accordingly, sorafenib, a ferroptosis activator, was shown to induce the death of cultured thyroid cancer cells [156,157].

### 3.4. Zinc

Zinc ranks as the second, after iron, most prevalent transition metal found in the human organism. It acts as a cofactor in a wide variety of metabolic metalloenzymes and is required for zinc-finger domain containing transcription factors. It participates in numerous cellular processes including the synthesis of proteins and DNA, antioxidant activity, wound healing, cellular division, and the proper functioning of the immune and nervous system [158]. It also plays a pivotal role in both the formation and metabolism of thyroid hormones. Accordingly, zinc acts as a cofactor of deiodinases DIO1 and DIO2, mediates the synthesis of thyrotropin-releasing hormone (TRH) in the hypothalamus and TSH in the pituitary gland, and is a cofactor in the zinc-finger domain of the transcription factor TTF-2 (Forkhead Box E1, FOXE1), which is one of the major transcription factors regulating the thyroid expression of TG, TPO, and NIS. Last but not least, zinc is a component of the zinc-finger domains in T3 nuclear receptors in all TH-responsive tissues [159]. Moreover, interestingly, one of the zinc-finger domain-containing transcription factors is GLI3 (GLI-similar 3), which controls the expression of genes involved in TH metabolism including *NIS*, *PDS*, *DUOX2*, *TPO*, *TG*, and *DIO1*. Consequently, GLI3 gene mutations lead to congenital neonatal hypothyroidism [160].

Zinc is required for DNA integrity as it is involved in the anti-oxidative stress response as a cofactor of copper-zinc superoxide dismutases (Cu/Zn SODs), namely SOD1 and SOD3, KEAP1 as well as metallothioneins [158,161]. Zinc is also required for, or modulates, the activity of multiple DNA repair proteins (Table 2) [158,161,162,163]. Moreover, zinc is also a component of chromatin remodelling complexes, such as histone deacetylases (HDACs), histone acetyltransferases (HATs), and histone demethylases, which play a critical role in regulating DNA accessibility for repair and transcription [164].

In non-thyroidal tissues or cell types, both zinc deficiency and overload lead to increased DNA damage, suggesting a similar impact on the genomic integrity of thyroid cells [179,180,181,182]. However, direct evidence of the influence of zinc level on thyroid DNA integrity is still missing. Decreased zinc levels are often associated with hypothyroidism and thyroid autoimmunity, thyroid nodules, and thyroid cancer [20,183,184]. Nevertheless, a systematic review analysing the influence of zinc supplementation on TH levels provided inconclusive results [185]. Moreover, a recent Mendelian randomisation study failed to provide evidence for zinc deficiency in TC patients [186]. Importantly, zinc also has immunomodulatory properties. Therefore, both zinc deficiency and excess can dysregulate immune responses, potentially influencing the development and progression of AITD as well as promoting the immune escape of TC cells. Specifically, zinc deficiency skews the Th1/Th2 and Treg/Th17 balance towards the Th2 and Th17 profile, thus potentially supporting autoimmunity and cancer immune escape [187,188].

### 3.5. Copper

Copper, the third most abundant transition metal, is also implicated in DNA stability through its role as a cofactor of Cu/Zn SODs 1 and 3 as well as ETC cytochrome C oxidase. Copper also mediates iron homeostasis through its involvement in active sites of the iron oxidising enzymes ferroxidases, especially the major plasmatic Cu carrier ceruloplasmin. It also modulates ferroptosis, a specific subtype of programmed cell death involving iron. As a transition metal, Cu participates in Fenton-like reactions, thereby contributing to ROS generation and leading to DNA damage, especially upon overload. Excessive Cu levels induce copper-dependent cell death, cuproptosis, which leads to the aggregation of critical lipoylated proteins involved in the mitochondrial TCA cycle as well as the loss of iron-sulphur clusters. This results in proteotoxic stress and metabolic dysfunction and culminates in cell death [189,190,191,192]. Copper also directly interferes with DNA repair, as evidenced by observation of the Cu-mediated inhibition of PARP-1, ALKBH2, ALKBH3, NEIL1 and NEIL2, and MTH1 as well as single-strand DNA break repair kinase/phosphatase PNKP [193,194,195,196,197].

Similarly to other cell systems, maintaining the physiological copper levels supports thyroid health including thyroid DNA integrity. Specifically, the serum copper concentration correlates with the thyroid hormone levels [198,199]. Moreover, Cu is essential for thyroid function as it participates in TH production and its regulation [20,184]. Elevated copper concentrations have been detected in the serum samples of AITD patients, however, not all studies have confirmed this association [20,88,184,199]. Increased serum levels of Cu were also observed in patients with nodular goitre and TC [20,184,200]. Additionally, the expression of cuproptosis-related genes was dysregulated in TC [201,202]. Copper also supports BRAF^V600E^-mediated tumorigenesis and consequently, Cu chelation inhibits the growth of BRAF-mutated PTCs in the murine model [203,204]. Preclinical studies suggest that therapies depleting copper or utilising copper-based agents that induce ROS-driven cell death and/or cuproptosis are promising approaches in the treatment of thyroid cancer [20,184,205,206]. Interestingly, systemic copper deficiency or overload, as observed in congenital disorders of Cu homeostasis such as Menkes’ and Wilson’s diseases, impairs double-strand DNA break repair and consequently disrupts the cellular response to radiation exposure in skin fibroblasts [207]. Therefore, it has to be considered that unbalanced copper levels may possibly affect the response to radioiodine-based agents in the diagnosis and/or therapy of TC or hyperthyroid patients.

### 3.6. Vitamin D

Vitamin D (VD) is a general name referring to the group of five secosteroids (vitamins D1–D5) [21]. In the context of thyroid health, the most relevant is vitamin D3 (cholecalciferol), which is synthesised in the skin in response to sunlight exposure. The active form of vitamin D3 is 1-α-25-dihydroxyvitamin D3 (calcitriol), which is synthesised from 25-hydroxyvitamin D (calcidiol, a.k.a. calcifediol, 25(OH)D) in the kidneys. Several meta-analyses have demonstrated the associations between decreased serum concentrations of 25(OH)D and AITD, particularly in Hashimoto’s thyroiditis [21]. These findings are in line with the observations of the beneficial effects of VD supplementation in animal models of AITD. In contrast, the links between VD deficiency and TC in clinical reports are less clear, although in vitro and in vivo studies suggest the anti-proliferative and anti-metastatic effects of VD supplementation [21,208,209]. In the context of DNA damage, VD has been suggested to modulate oxidative stress and protect against DNA lesions as well as stimulate DNA repair [210,211,212,213]. However, direct evidence of vitamin D involvement in protection against DNA damage or in DNA repair in the context of thyroid disease is still missing. The role of vitamin D in thyroid health and disease was extensively discussed in an excellent review by Babić Leko et al. [21].

### 3.7. Folate (Vitamin B9) and Vitamin B12

**Figure 3:**
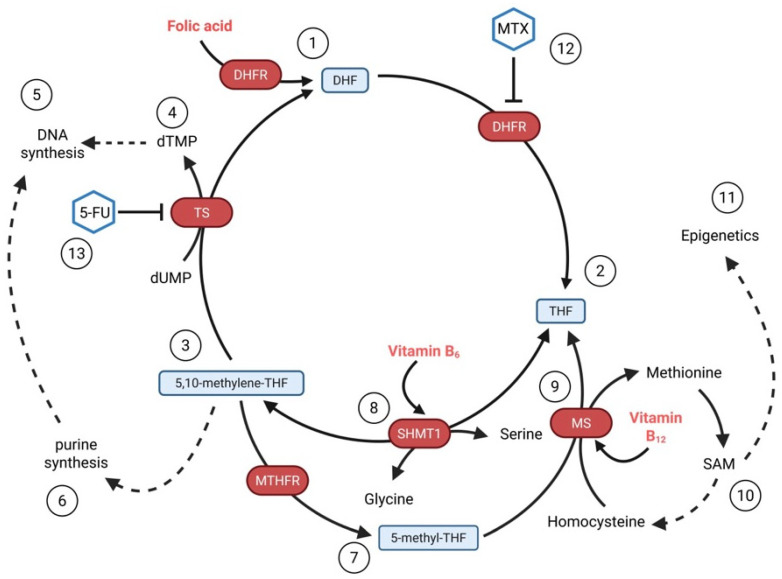
Overview of One-Carbon Metabolism. One carbon metabolism, which includes the folate and methionine cycles, involves (**1**) reduction of folic acid by dihydrofolate reductase (DHFR) to dihydrofolate (DHF) and then to (**2**) tetrahydrofolate (THF). THF is a central molecule in this pathway, involved in the synthesis of (**3**) 5,10-methylene-THF, which is crucial for (**4**) deoxythymidylate (dTMP) synthesis (via thymidylate synthase, TS) and ultimately (**5**) DNA synthesis. 5,10-Methylene-THF also serves (**6**) as a one-carbon unit donor in de novo purine synthesis and is converted to (**7**) 5-methyl-THF by methylenetetrahydrofolate reductase (MTHFR). (**8**) Serine hydroxymethyltransferase 1 (SHMT1), assisted by vitamin B_6_, interconverts serine and glycine simultaneously, interconverting THF and 5,10-methylene-THF. (**9**) Methionine synthase (MS), aided by vitamin B_12_, regenerates methionine from homocysteine with the help of a methyl group donated by 5-methyl-THF that is simultaneously converted to THF. Methionine can be further converted to (**10**) S-adenosylmethionine (SAM), which is a universal methyl group donor in multiple biological processes including the (**11**) methylation of histones and cytosine residues in DNA. One-carbon metabolism enzymes are targets for chemotherapeutics: (**12**) methotrexate (MTX) inhibits DHFR, and (**13**) 5-fluorouracil (5-FU) inhibits TS. Red font: micronutrients. Created in BioRender. Arczewska, K. (2025) https://BioRender.com/0x2ez7c (accessed on 16 June 2025).

Folate (FA; vitamin B9) is an important nutrient in the context of genomic DNA stability, predominantly through its requirement for the synthesis of purines and pyrimidines in the one-carbon metabolism pathway (Figure 3). In particular, pyrimidine synthesis involving the conversion of deoxyuridine monophosphate (dUMP) to deoxythymidine monophosphate (dTMP) by thymidylate synthase requires folate metabolites. Vitamin B12 is also an important cofactor in one-carbon metabolism, required by methionine synthase to generate methionine and tetrahydrofolate, a folate derivative required for downstream metabolic reactions. Moreover, folate and vitamin B12 contribute to the production of S-adenosylmethionine (SAM), the universal methyl donor, essential for the methylation of histones and cytosine residues in DNA, which constitutes the core of the epigenetic control of gene expression [214]. Therefore, folate and vitamin B12 levels influence DNA replication, repair, and methylation. Specifically, folate and vitamin B12 deficiency may lead to elevated levels of deoxyuridine triphosphate (dUTP) in the nucleotide pool, followed by genomic uracil incorporation and the removal of uracil residues by uracil DNA glycosylase (UNG)-initiated BER, which can result in the accumulation of DNA strand breaks [215,216].

Folate derivative-requiring enzymes are the targets of two widely utilised chemotherapeutics, namely 5-fluorouracil (5-FU), an inhibitor of thymidylate synthase, and methotrexate (MTX), an inhibitor of dihydrofolate reductase (DHFR) that synthesises tetrahydrofolate [214]. Combining the fluoropyrimidine 5-FU with other chemotherapeutics was proposed for the management of metastatic medullary thyroid cancer, but this approach did not result in better clinical outcomes [217]. MTX has been explored as a part of multi-drug chemotherapy regimens in aggressive or advanced thyroid cancers resistant to conventional therapeutic treatments. Recently, targeted delivery approaches have been investigated to target methotrexate to thyroid cancer cells, for example, using nanoparticle carriers [218]. MTX, on the other hand, also has immunosuppressive activity and therefore can be utilised in combination with glucocorticosteroids in the management of thyroid eye disease/Graves’ orbitopathy [219]. In patients with hypothyroidism and AITD, folate and/or vitamin B12 deficiency is observed, and might be associated with anaemia [141,220]. Interestingly, vitamin B12 was the only one among the tested 15 micronutrients, including iron, selenium, zinc, copper, folate and vitamin D, that showed an association with genetic susceptibility to autoimmune thyroiditis [221].

## 4. Environmental Factors

The lifestyle or environmental factors that influence thyroid DNA integrity include, among others, radiation, cigarette smoking, and endocrine disruptors. Perhaps the most extensively studied among these environmental insults is radiation exposure, since it exerts a detrimental impact on thyroid health. As can be learned from studies involving nuclear accident survivors, especially those exposed to irradiation during childhood, radiation leads to an increased incidence of thyroid disease including thyroid nodules, follicular adenoma, subclinical hypothyroidism, and AITD as well as PTC with an increased frequency of DNA double-strand break-induced *RET::PTC* and other rearrangements [222,223,224,225,226]. The increased incidence of thyroid carcinogenesis after radiation exposure during childhood is most likely related to the fact that in young individuals, thyrocytes actively divide and thus show elevated levels of IR-induced DNA damage in comparison to the stationary thyrocytes in adults [227]. Furthermore, several studies have suggested that diagnostic X-ray exposure may be associated with an increased risk of thyroid cancer [228,229,230]. On the other hand, according to the recent recommendations of the ADA (American Dental Association), thyroid and abdominal shielding during dental imaging is no longer recommended [231].

Cigarette smoke affects thyroid physiology, mainly through the thiocyanate-mediated inhibition of NIS-dependent iodine transport. This may interfere with the TH levels, leading to thyrocyte iodide deficiency and the resulting oxidative stress and DNA damage [32,140]. Consequently, the prevalence of thyroid disease, including GD, HT, thyroid nodules, and goitre, is increased among smokers. However, surprisingly, a reduced TC incidence among smokers is repeatedly observed [232]. Importantly, however, smoking increases the TC incidence in carriers of cancer risk-associated polymorphisms in several DNA repair-related genes [233,234,235].

Endocrine disruptors (EDs) are industrial or agricultural substances that interfere with the endocrine system function. Apart from their endocrine disturbing activity, EDs may potentially generate DNA adducts, inhibit DNA repair processes, induce epigenetic reprograming, and interfere with DNA repair protein expression, thus contributing to mutagenesis, carcinogenesis, and cancer cell chemoresistance [236,237,238,239,240]. In the thyroid, EDs have been suggested to interfere with thyroid function and contribute to thyroid disorders including thyroid nodules, TC, and AITD. For example, the bisphenol A (BPA) plasticiser has been extensively studied in the context of thyroid disease [140,237,241,242,243]. BPA toxicity and carcinogenic potential is mediated, at least partially, through the induction of oxidative stress, accompanied by DNA damage and the inhibition of DNA repair [244]. Moreover, BPA treatment interferes with DNA repair-related processes, including downregulation of the expression of base excision repair (BER) pathway components [242,245,246]. Interestingly, BPA treatment of rat thyrocytes interfered with iodide uptake as well as the Nis and Duox mRNA levels, leading to increased H_2_O_2_ generation and OS induction as well as inhibition of the DNA damage response [247,248]. Another group of industrial chemicals that are considered EDs are per- and polyfluoroalkyl substances (PFAS). Humans are exposed to PFAS through dietary ingestion due to their wide distribution in soil and water as well as their usage in food packaging materials. Importantly, PFAS as EDs interfere with TH homeostasis [249]. Moreover, PFAS exposure has been described to increase the TC risk [250]. However, until now, limited studies have described PFAS interference with genome stability, and sparse reports have described the PFAS genotoxic activity mainly through the induction of oxidative stress, but also through cGAS induction followed by the inhibition of DNA double strand break repair [251,252,253].

## 5. Conclusions

This review highlights that both micronutrient imbalances and environmental stressors can compromise DNA integrity in thyroid cells, potentially contributing to autoimmune thyroid disease and thyroid cancer. Disrupted antioxidant defence and DNA repair mechanisms appear central to this process. Maintaining the optimal micronutrient levels and reducing the exposure to harmful agents, such as radiation and endocrine disruptors, is essential. Further studies are needed to assess the clinical value of selenium and zinc supplementation, the risks associated with frequent diagnostic radiation, and the impact of endocrine-disrupting chemicals on thyroid genome stability.

## Figures and Tables

**Table 1:** The iron-containing enzymes implicated in DNA damage prevention, DNA metabolism, replication, and stability.

Name of Enzyme/Gene	Primary Function	Iron Cofactor	Involvement in Thyroid Disease	References
Catalase	Removes H_2_O_2_ by its hydrolysis to water and oxygen	Heme iron centre	Downregulated in TC, GD, HT, and follicular adenoma (FA) tissues.	[55,99,100,101,102]
Ribonucleotide reductase (RRM2 subunit)	Synthesis of deoxyribonucleotides (dNTPs)	Di-iron centre	RRM1 subunit is overexpressed in PTC; RRM2 subunit is overexpressed in PTC, TC, and ATC tissues. RRM1 and RRM2 overexpression positively correlates with markers of aggression, and disease progression.	[103,104,105]
Dihydropyrimidine dehydrogenase (DPYD)	Pyrimidine catabolism. Brakes down uracil, thymine and 5-FU	Fe-S cluster	*DPYD* expression is more pronounced in PTC than in normal tissues, which may suggest a poor response to 5-FU-based therapies.	[106]
Phosphoribosyl pyrophosphate amidotransferase (PPAT)	De novo purine synthesis pathway	Fe-S cluster	*PPAT* is overexpressed in TC and supports malignant traits.	[107]
Xanthine dehydrogenase/oxidase	Purine catabolism. The enzyme exists in two forms: xanthine dehydrogenase (XDH) and xanthine oxidase (XO).	Fe-S cluster	XDH/XO levels are in general low in thyroid tissue but upregulated in TC.	[108]
DNA polymerase α (POLA)	Initiation of DNA replication. Modulation of interferon responses.	Fe-S cluster	POLA1 catalytic subunit defect may lead to developmental delay without the thyroid phenotype. Another medical condition associated with POLA1 deficiency is autoinflammatory interferonopathy, with a lack of signs of autoimmune disease including lack of AITD. The TCGA dataset analysed using the UALCAN cancer database (https://ualcan.path.uab.edu, accessed on 23 April 2025) suggests the downregulation of *POLA1* in TC tissues.	[109,110,111,112]
DNA polymerase δ (POLD)	DNA replication and repair. Involved in multiple DNA repair pathways.	Fe-S cluster	Catalytic subunit *POLD1* is downregulated in PTC and associated with poor clinical course. *POLD1* mutations in benign thyroid goitre and PTC imply involvement in cancer progression.	[113,114]
DNA polymerase ε (POLE)	DNA replication and repair. Involved in multiple DNA repair pathways.	Fe-S cluster	POLE catalytic subunit is downregulated in PTC tissues. Deficiency of accessory POLE subunit (*POLE2*) induces hypothyroidism.	[113,115]
DNA polymerase ζ (POLZ)	Translesion DNA synthesis	Fe-S cluster	TCGA dataset analysed using the UALCAN cancer database (https://ualcan.path.uab.edu) suggests the downregulation of POLZ catalytic subunit *REV3L* and the upregulation of accessory subunit *REV7/FANCV/MAD2L2* in TC tissues	[110,111,112,116]
DNA primase (PRIM)	Initiation of RNA primers for DNA replication	Fe-S cluster	TCGA dataset analysed using the UALCAN cancer database (https://ualcan.path.uab.edu) suggests a slight downregulation of catalytic, PRIM1, and regulatory PRIM2 subunits in TC tissue.	[110,111,112,117]
Helicase XPD/ERCC2	Nucleotide excision repair, transcription initiation	Fe-S cluster	*XPD* polymorphisms may increase TC susceptibility in general, as well as increase radiation-related TC risk. Low XPD expression is associated with BRAF^V600E^ mutation and markers of aggressive disease.	[118,119,120,121,122,123]
Helicase FANCJ/BRIP1/BACH1	Double-strand break repair, interstrand crosslink repair (Fanconi anaemia pathway)	Fe-S cluster	*FANCJ* polymorphisms are not associated with TC risk.	[120,124,125]
Helicase DNA2	Okazaki fragment processing, DNA repair, telomere maintenance	Fe-S cluster	*DNA2* is frequently deleted and overexpressed in TC.	[126]
Helicase RTEL1	Telomere maintenance	Fe-S cluster	*RTEL1* is upregulated in TC.	[127]
Helicase DDX11/ChlR1	Involved in homologous recombination and tolerance of replication stress	Fe-S cluster	*DDX11* shows a low expression level in thyroid tissue without expression changes in TC.	[128]
Exonuclease EXO5	UV-induced DNA damage and interstrand crosslink repair	Fe-S cluster	*EXO5* is downregulated in TC tissue.	[129]
Glycosylase NTH1/NTHL1	Base excision repair; repairs oxidised pyrimidines, mainly Tg, 5-OHC, 5-OH	Fe-S cluster	Increased incidence of TC in *NTH1* mutation carriers. NTH1 polymorphisms have no influence on radiation related TC risk.	[125,130]
Glycosylase MUTYH	Base excision repair (repairs A:G and A:8-oxoG mismatches)	Fe-S cluster	*MUTYH* mutation increases the thyroid nodule frequency and PTC risk. In thyroid cells, *MUTYH* is upregulated upon oxidative stress.	[131,132,133]
Demethylase ALKBH2/3	Direct repair of alkylated DNA bases	Non-heme iron centre	ALKBH3 polymorphisms may increase TC susceptibility in general as well as increase the radiation-related TC risk.	[125,134]

5-FU—5-fluorouracil; FA—follicular adenoma; GD—Graves’ disease; HT—Hashimoto thyroiditis; TC—thyroid cancer; RRM1—ribonucleotide reductase catalytic subunit M1; RRM2—ribonucleotide reductase regulatory subunit M2.

**Table 2:** The zinc-containing enzymes implicated in DNA repair.

Name Enzyme/Gene	Primary Function	Role of Zinc	Involvement in Thyroid Disease	References
Endonuclease APE1/APEX1/Ref-1	Single-strand DNA break repair; involved in redox signalling	May act as a cofactor; modulates activity	APE1/Ref-1, in addition to its involvement in DNA repair, also acts as a redox regulator modulating DNA-binding and the transcriptional activity of thyroid-specific transcription factors Pax8 and TTF-1 in thyroid cells. APE1 expression is elevated in thyroid cancer tissues and in the nuclear fractions of hyperfunctioning thyroid nodules. Inhibiting the redox domain of APE1 has shown promise in overcoming resistance to certain cancer therapies in preclinical models.	[165,166,167]
Endonuclease APE2/APEX2	Single-strand DNA break repair	Cofactor in zinc finger domain	TCGA dataset analysed using the UALCAN cancer database (https://ualcan.path.uab.edu) suggests the *APE2* upregulation in TC tissue.	[110,111,112]
APTX	Single-strand DNA break repair	Cofactor in zinc finger domain	TCGA dataset analysed using the UALCAN cancer database (https://ualcan.path.uab.edu) suggests the downregulation of APTX in TC tissue.	[110,111,112]
BARD1	Double-strand DNA break repair	Cofactor in zinc finger domain	TCGA dataset analysed using the UALCAN cancer database (https://ualcan.path.uab.edu) suggests the downregulation of *BARD1* in TC tissue.	[110,111,112]
BRCA1/FANCS	Double-strand DNA break repair, interstrand crosslink repair (Fanconi anaemia pathway)	Cofactor in zinc finger domain	*BRCA1* genetic variation may modulate TC risk.	[16]
Endonuclease CtIP/RBBP8	Double-strand DNA break repair	Cofactor in zinc finger domain	TCGA dataset analysed using the UALCAN cancer database (https://ualcan.path.uab.edu) suggests the downregulation of *CtIP* in TC tissue.	[110,111,112]
Ligase LIG3α	Base excision repair; double-strand DNA break repair	Cofactor in zinc finger domain	TCGA dataset analysed using the UALCAN cancer database (https://ualcan.path.uab.edu) suggests the downregulation of *LIG3α* in TC tissue.	[110,111,112]
MDM2	DNA damage response	Cofactor in zinc finger domain	*MDM2* is overexpressed in TC tissues. *MDM2* genetic variation increases TC risk.	[168,169]
MDM4	DNA damage response	Cofactor in zinc finger domain	*MDM4* is often downregulated in TC tissue.	[168]
Glycosylase MUTYH	Base excision repair	Cofactor in Zinc Linchpin Motif	*MUTYH* mutation increases thyroid nodule frequency and PTC risk. In thyroid cells, *MUTYH* is upregulated upon oxidative stress.	[131,132,133]
Glycosylase NEIL2	Base excision repair	Cofactor in zinc finger domain	TCGA dataset analysed using UALCAN cancer database (https://ualcan.path.uab.edu) suggest downregulation of *NEIL2* in TC tissue.	[110,111,112]
Glycosylase NEIL3	Base excision repair	Cofactor in zinc finger domain	*NEIL3* polymorphisms may modulate TC risk	[170]
Glycosylase OGG1	Base excision repair	Cofactor in zinc finger domain	*OGG1* polymorphisms are associated with increased GD but not TC risk. OGG1 is overexpressed in PTC tissues.	[99,171,172]
PARP1	Single-strand DNA break repair; double-strand DNA break repair; DNA damage response	Cofactor in zinc finger domain	Application of PARP1 inhibitors was suggested in TC management.	[173]
Polymerase POLE	DNA replication and repair. Involved in multiple DNA repair pathways.	Cofactor in ubiquitin-binding zinc finger	POLE catalytic subunit is downregulated in PTC tissues. Deficiency of accessory POLE subunit (*POLE2*) induces hypothyroidism.	[113,115]
RAD18	Translesion synthesis	Cofactor in zinc finger domain	RAD18 gene expression is elevated in PTC tissues harbouring the BRAF^V600E^ mutation. Increased RAD18 levels are positively associated with poor prognosis in these cases.	[174]
RAD50	Double-strand DNA break repair	Cofactor in zinc hook domain	Germline *RAD50* variants may increase TC risk.	[175]
RPA	RPA protects binds single-stranded DNA from nucleolytic degradation. Involved in multiple DNA repair pathways	Cofactor in zinc finger domain	Germline variants of RPA subunits may increase susceptibility to TC. TCGA dataset analysed using UALCAN cancer database (https://ualcan.path.uab.edu) suggest downregulation of *RPA* subunits in TC tissue.	[110,111,112,176]
TP53	DNA damage response	Cofactor in DNA-binding core domain	*TP53* mutations are frequent in ATC tumours and are associated with dedifferentiation and disease progression. *TP53* polymorphisms may increase TC risk. TP53 reactivation may support anti-tumour immune responses in TC patients and suppress autoimmunity observed in AITD.	[16,177,178]
XPA	Nucleotide excision repair	Cofactor in zinc finger domain	TCGA dataset analysed using the UALCAN cancer database (https://ualcan.path.uab.edu) suggests the downregulation of *XPA* in TC tissue.	[110,111,112]

ATC—anaplastic thyroid cancer; GD—Graves’ disease; PTC—papillary thyroid cancer; TC—thyroid cancer.
